# The Earliest Colubroid-Dominated Snake Fauna from Africa: Perspectives from the Late Oligocene Nsungwe Formation of Southwestern Tanzania

**DOI:** 10.1371/journal.pone.0090415

**Published:** 2014-03-19

**Authors:** Jacob A. McCartney, Nancy J. Stevens, Patrick M. O’Connor

**Affiliations:** 1 Department of Anatomical Sciences, Health Sciences Center, Stony Brook University, Stony Brook, New York, United States of America; 2 Department of Biomedical Sciences, Irvine Hall, Heritage College of Osteopathic Medicine, Ohio University, Athens, Ohio, United States of America; 3 Center for Ecology and Evolutionary Studies, Irvine Hall, Ohio University, Athens, Ohio, United States of America; University of Birmingham, United Kingdom

## Abstract

The extant snake fauna has its roots in faunal upheaval occurring across the Paleogene - Neogene transition. On northern continents, this turnover is well established by the late early Miocene. However, this transition is poorly documented on southern landmasses, particularly on continental Africa, where no late Paleogene terrestrial snake assemblages are documented south of the equator. Here we describe a newly discovered snake fauna from the Late Oligocene Nsungwe Formation in the Rukwa Rift Basin of Tanzania. The fauna is small but diverse with eight identifiable morphotypes, comprised of three booids and five colubroids. This fauna includes *Rukwanyoka holmani* gen. et sp. nov., the oldest boid known from mainland Africa. It also provides the oldest fossil evidence for the African colubroid clade Elapidae. Colubroids dominate the fauna, comprising more than 75% of the recovered material. This is likely tied to local aridification and/or seasonality and mirrors the pattern of overturn in later snake faunas inhabiting the emerging grassland environments of Europe and North America. The early emergence of colubroid dominance in the Rukwa Rift Basin relative to northern continents suggests that the pattern of overturn that resulted in extant faunas happened in a more complex fashion on continental Africa than was previously realized, with African colubroids becoming at least locally important in the late Paleogene, either ahead of or as a consequence of the invasion of colubrids. The early occurrence of elapid snakes in the latest Oligocene of Africa suggests the clade rapidly spread from Asia to Africa, or arose in Africa, before invading Europe.

## Introduction

The origin of the Recent snake fauna occurred in the early Miocene, when ancient snake faunas dominated by basal snakes gave way to colubroid dominated communities [1]–[3]. This transition may be tied to a drying climate, one that resulted in a shift from the closed habitats that favor sit-and-wait ambush predators to more open habitats interpreted to favor active predators [4]. This pattern of faunal turnover is well documented in Europe [5]–[11] and North America (e.g., [1], [2], [12], [13]–[15]) as a late early Miocene invasion from Asia.

Fossil evidence suggests that the colubroid radiation was underway by the late Eocene, with representatives reported in Europe [16]–[19], North America [20], [21], Africa [22], and Asia [23]–[26]. It is even possible that colubroid evolutionary history extends into the Late Cretaceous [27], although Head and colleagues [23] have cautioned that this should be regarded as a tentative scenario. Colubroids remained rare in North America and Europe through the Oligocene [28]–[31].

Molecularly derived divergence estimates using fossils as calibration points suggest that Colubroidea appeared no later than the Late Cretaceous, which is congruent with fossil specimens of possible colubroid snakes from the Cenomanian of Sudan [27]. Similarly derived divergence estimates predict the radiation of colubroids into modern subfamilies as early as the Eocene [32]–[37], yet fossil evidence has been limited Oligocene-to-later elapids from Australia, Europe and North America (e.g., [14], [38], [39], [40]), and a Plio-Pleistocene record of the extant psammophiine *Malpolon* in Europe [41], [42].

To date, our understanding of evolutionary patterns in the Paleogene of continental Africa is largely gleaned from circum-Saharan faunas that consist of assemblages dominated by basal alethinophidian snakes [43]–[46]. With the exception of a single vertebra from the Eocene of Namibia [22], the terrestrial snake record from the southern part of the continent is derived from Neogene-aged rocks, with caenophidian-dominated faunas known from what is now Kenya [47]–[49], Uganda [50], Tanzania [51], [52], Namibia [53], and South Africa [54]. Although isolated occurrences of aquatic snakes appear in Paleogene marine deposits in southern Africa [55]–[58], the terrestrial snake record is notably absent. As a result, it has been impossible to document whether continental Africa was characterized by an early Miocene transition from faunas dominated by basal alethinophidians to caenophidian-dominated assemblages as is the case in the northern continents.

Field research conducted in the Rukwa Rift Basin of southwestern Tanzania is beginning to address important gaps in the Cenozoic terrestrial and freshwater vertebrate record of Africa. In particular, recent work in the Nsungwe Formation has revealed a diverse fauna of late Oligocene age, preserving invertebrates [59], fish [60], anurans [61], crocodylians, and mammals [62]–[66]. Here we describe the first terrestrial snake assemblage from the Paleogene of southern Africa that includes the first occurrences of several snake clades and documents the transition to caenophidian-dominated faunas in the region.

### Geologic Setting

**Figure 1:**
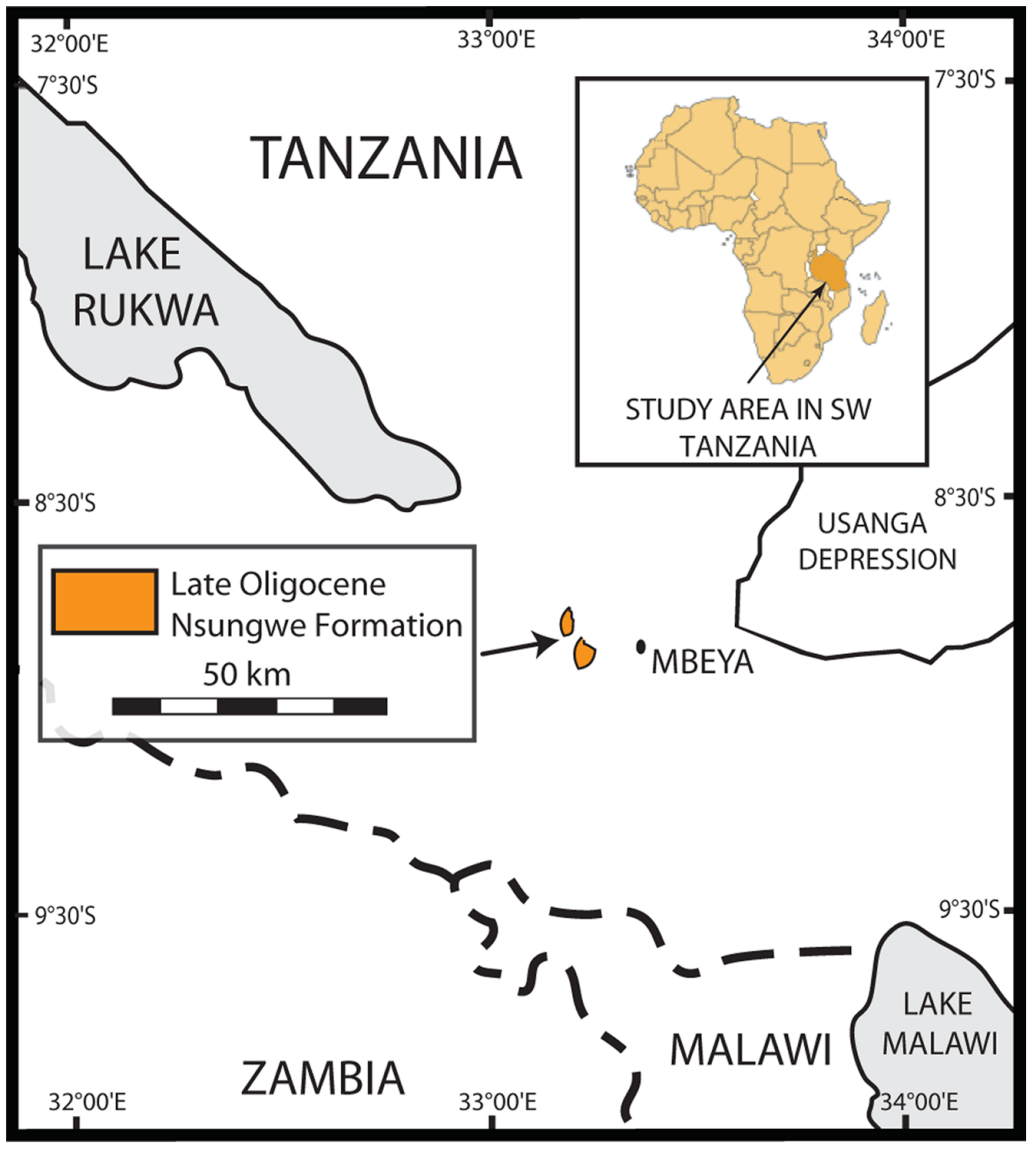
Geologic setting and outcrop area of the Nsungwe Formation in the Rukwa Rift Basin of southwestern Tanzania.

The study area is situated in the Rukwa Rift Basin of southwestern Tanzania (Fig. 1). Fossils were excavated from outcrops in the late Oligocene Nsungwe Formation. These deposits represent a continental rift-fill sequence containing a number of recently discovered fossil-bearing localities in different temporal intervals [65], [67], [68]. The Nsungwe Formation is subdivided into the lower Utengule Member and upper Songwe Member, with the snake-bearing Songwe Member assigned a Late Oligocene (∼24.95 MY) age based on biostratigraphy, dated ash beds, and detrital zircon geochronology [62]–[65], [68]–[72].

Fossils described herein were discovered in fluvial facies at localities TZ-01, TZ-01S, TZP-2, and Nsungwe 2, four of the richest localities of the Songwe Member of the Nsungwe Formation. Facies associations and faunal data, including the presence of aquatic and semi-aquatic taxa (fish, frogs, crustaceans, and molluscs), suggest perennial availability of water with periodic or seasonal climatic fluctuation [72].

## Methods

### Nomenclatural Acts

The electronic edition of this article conforms to the requirements of the amended International Code of Zoological Nomenclature, and hence the new names contained herein are available under that Code from the electronic edition of this article. This published work and the nomenclatural acts it contains have been registered in ZooBank, the online registration system for the ICZN. The ZooBank LSIDs (Life Science Identifiers) can be resolved and the associated information viewed through any standard web browser by appending the LSID to the prefix “http://zoobank.org/”. The LSID for this publication is: urn:lsid:zoobank.org:pub:308EF90E-CEF3-45B2-AA31-FA3C1E698692. The electronic edition of this work was published in a journal with an ISSN, and has been archived and is available from the following digital repositories: PubMed Central, LOCKSS.

### Permits

All necessary permits were obtained for the described study, which complied with all relevant regulations. Fieldwork was conducted under permits issued by the Tanzanian Commission for Science and Technology (COSTECH), the Tanzania Antiquities Unit, and the Tanzanian Division of Immigration.

### Specimen Preparation

**Figure 2:**
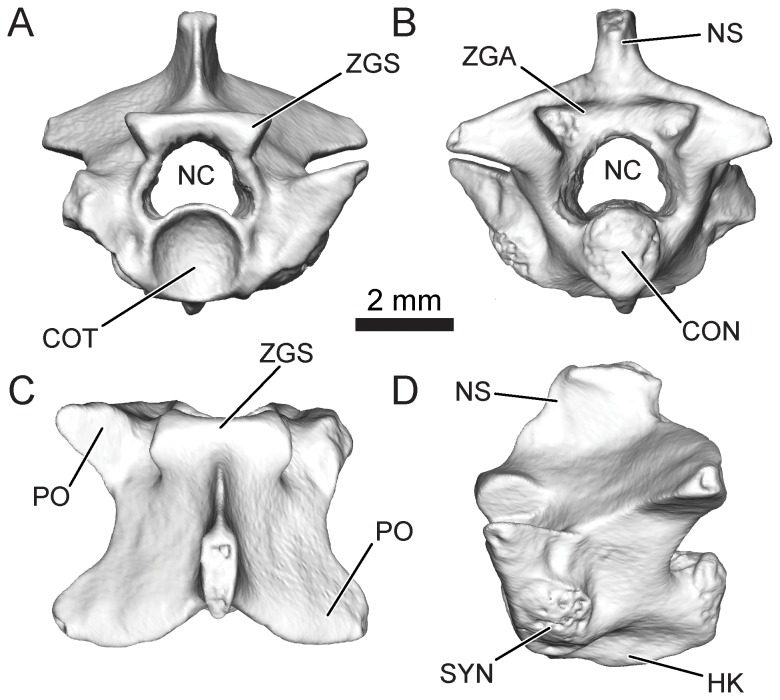
Basic snake vertebral morphology. Four views of mid-trunk vertebra of the boine boid *Rukwanyoka holmani* (RRBP 10041). A, Anterior view; B, Posterior view; C, Dorsal view; D, Left lateral view. CON, condyle; COT, cotyle; HK, hemal keel; NC, neural canal; NS, neural spine; PO, prezygapophysis; PR, postzygapophysis; SYN, synapophis; ZGA, zygantrum; ZGS, zygosphene.

Subsequent to mechanical preparation at the Ohio University Fossil Preparation and Imaging Facility specimens were photographed on a Nikon stereomicroscope bundled with Spot Advanced (version 3.5) software. Calibrated measurements were made on these images using ImageJ v. 10.2 [73]. The accuracy of measurements is less than +/−0.1 mm. Representative vertebrae were also scanned using a GE eXplore Locus micro-computed tomography scanner (GE Healthcare Pre-Clinical Imaging, London, ON, Canada) housed at Ohio University. Scans were acquired at an x-ray tube voltage of 80 kV, a current of 450 μA, and an effective voxel size of 0.045mm. Post-scan processing and visualization were completed in Avizo 6.3 (VSG-Visualization Sciences Group/FEI, United States). Basic snake vertebral morphology and the terms used throughout this contribution are illustrated in Fig. 2. Comparative material consisted of reference specimens and photographs of representative fossil and Recent snakes derived from collections housed at Carnegie Museum of Natural History (CM), Museum of Comparative Zoology (MCZ), National Museum of Natural History (USNM), and Yale Peabody Museum (YPM), in addition to images published in the primary literature (for a full specimen list, please see Supporting Information S1).

### Note on Taxonomy

**Figure 3:**
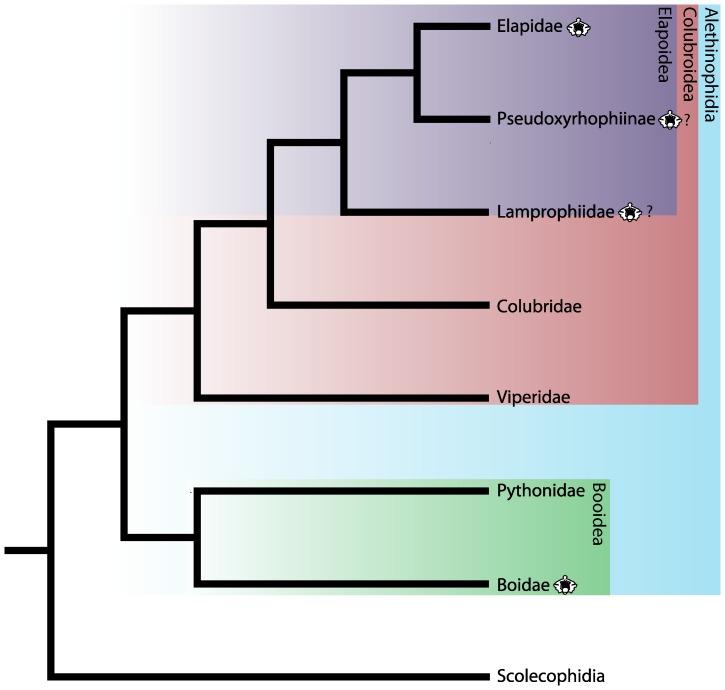
Phylogeny of major African snake clades. Topology after [32]. Note that in some studies Pseudoxyrhophiinae is recovered as a monophyletic clade within Lamprophiidae [88], [128], or that Lamprophiidae is paraphyletic with respect to Elapidae [37].

Snake systematics is currently in a state of flux, with a significant lack of congruence in topologies based on molecular and morphological datasets [32], [74]–[84]. Most differences occur in basal taxa not represented in the Nsungwe Formation. However, the situation is complicated in that few morphological analyses include a broad sample colubroid snakes. A recent exception [84] reveals considerable disparity in the morphological and molecular [85]–[88] hypotheses of caenophidian snake relationships. Yet the authors of that analysis were careful to note that their taxon sampling was not sufficiently dense to confidently establish caenophidian relationships. Accordingly, we utilize the phylogenetic framework of Pyron & Burbrink [32], as it represents the most taxonomically comprehensive phylogenetic analysis published to date (see Fig. 3 for a tree of major African clades). In that study, the Booidea consists of Cylindrophiidae, Boidae, Bolyeriidae, Pythonidae, and Uropeltidae, as well as the monotypic families Anomochilidae, Calabariidae, Loxocemidae, Xenopeltidae, and Xenophiidae. The traditional Booidea, consisting of Boidae, Pythonidae, Loxocemidae, Xenopeltidae, and Tropidophiidae, represents a paraphyletic assemblage in this taxonomy, as it is in most cladistic analyses [32], [74]–[76], [78]–[80], [83], [84], [89]–[91]. Colubroidea retains the traditionally broad definition of all Caenophidia exclusive of Acrochordidae.

## Results

### Systematic Hierarchy

Squamata Oppel, 1811 [92].

Serpentes Linnaeus, 1758 [93].

Alethinophidia Nopsca, 1923 [94].

Booidea Gray, 1825 [95].

Boidae Gray, 1825 [95].

Boinae Gray, 1825 [95].

*Rukwanyoka*, gen. nov.

urn:lsid:zoobank.org:act:8B20BEDF-0D07-4A72-BCBA-4080B35C36E4.

#### Type species

*Rukwanyoka holmani*, sp. nov.

#### Etymology

From Rukwa, the rift basin from which the fossils were recovered, and nyoka (Swahili), meaning snake.

#### Diagnosis

As for the type and only species.

*Rukwanyoka holmani*, sp. nov.

urn:lsid:zoobank.org:act:8B20BEDF-0D07-4A72-BCBA-4080B35C36E4.

**Figure 4:**
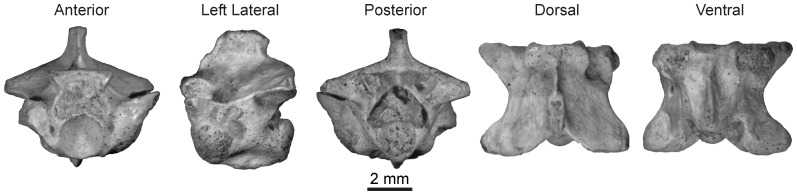
Mid-trunk vertebra of *Rukwanyoka holmani* (RRBP 10041) from the late Oligocene of Tanzania.

Figures 2, 4; Table 1; Supporting Information S2.

**Table 1:** Measurements (in mm) of the booid vertebrae in the collection.

Taxon	Specimen	CL	NAW	ZSW	CTW	CTH	CNW	CNH	PRW	POW	PrPo	NH	HH
*Rukwanyoka holmani*	10041	–	4.5	3.1	2.0	2.0	–	–	6.7	5.1	2.3	–	2.3
*Rukwanyoka holmani*	11543B	–	3.8*	2.6	1.8	1.4	–	–	–	–	–	–	–
Booid Morphotype B	11490	–	4.3	3.3	2.9	–	–	–	–	–	–	–	–
booid indet	04100	–	1.7	1.3	0.9	0.8	–	–	2.9	2.6	0.8	–	0.8
booid indet	04332	–	2.3	1.9	1.3	1.00	–	–	3.0	2.5	–	0.7	–
booid indet	06179	2.8*	3.6	–	2.0	1.5	–	–	–	–	3.4	–	–

**CL**, centrum length; **CNH**, condyle height; **CTH**, cotyle height; **CTW**, cotyle width; **HH**, hypapophyseal height; **NAW**, neural arch width; **NH**; neural spine height; **POW**, width across the postzygapophyses; **PrPo**, distance from the anterior edge of the prezygapophysis to the posterior edge of the postzygapophysis; **PRW**, width across the prezygapophyses; **ZSW**, zygosphene width.

*slight underestimates of values due to erosion or breakage.

#### Type specimen

Rukwa Rift Basin Project (RRBP–identifier used by the Tanzanian Antiquities Unit) 10041, mid-trunk vertebra.

#### Referred specimen

RRBP 11543B, mid- or posterior trunk vertebra.

#### Type locality

Late Oligocene Nsungwe Formation, locality Nsungwe 2, Rukwa Rift Basin, southwestern Tanzania.

#### Etymology

Named for J. Alan Holman in honor of his contributions to the field of snake paleontology.

#### Diagnosis

Distinguished from all other boids in having the following combination of features: neural spine longer than high, with thickened posterior portion and distinct thin, blade-like anterior margin; neural arch laminae straight; zygosphene gracile with flat superior surface and concave anterior surface; paracotylar foramina set high in deep paracotylar fossae; hemal keel deep and narrow in mid-trunk vertebrae.

#### Description

The holotype vertebra of *Rukwanyoka holmani* (RRBP 10041) is the largest snake vertebra recovered to date from the Nsungwe Formation fauna at 5.1 mm in length (Fig. 4; Table 1). It is short anteroposteriorly and relatively wide, with a strong waisting in dorsal view. The neural arch has straight laminae and a strongly notched posterior border. The nearly complete neural spine is moderately tall and anteroposteriorly long, with an anterior margin equivalent with the midpoint of the zygosphenal facets. It is thick posteriorly, but tapers abruptly to a thin anterior blade. In lateral view the blade is notched such that the anterior edge has a stepped appearance (Fig. 4). The spine is oriented nearly vertically such that there is only a weak posterior projection dorsal to the zygantrum. The zygosphene is short dorsoventrally, and thus exhibits facets that are in part positioned ventral to the level of the roof of the neural canal. The dorsal margin is flat with a weakly concave anterior edge that tapers to a thin ridge between the facets. The zygantrum is deep anteroposteriorly and short dorsoventrally, so that the roof of the neural canal separates the ventrolateral parts of the cavity. The zygantral cavities are confluent dorsally and roofed by the neural arch and each bears a single intrazygantral foramen in the anterior wall. The facets are orbicular and do not project posteriorly past the margin of the neural arch. The neural canal is large, tall, and subtriangular in shape.

The zygapophyses are represented in RRBP 10041 by both postzygapophyses but only one intact prezygapophysis. The left prezygapophysis bears an oval facet and is not laterally produced (sensu [96]). The prezygapophyseal accessory process is broken in both specimens. The postzygapophyses bear subtriangular facets. The weak expansion evident superior to each facet is suggestive of an epizygapophyseal spine, although the lateral extent of this process is broken. The pre- and postzygapophyses are dorsolaterally inclined at ∼ 75° from the median sagittal plane.

The centrum is relatively complete, although the synapophyses and condyle are incompletely preserved on both specimens. The cotyle is circular, and the paracotylar fossae are deep, each with a single large foramen positioned superior to cotylar mid-height. On the ventral surface of the centrum the hemal keel is tall and gladiate in appearance (sensu [97]). The hemal keel does not project as strongly immediately posterior to the cotyle. The subcentral fossae each bear a single, anteriorly situated subcentral foramen. The subcentral ridges are low, broad, and angled posteromedially.

The second specimen of this morphotype (RRBP 11543B) is serially positioned near the transition between the mid-trunk and the posterior trunk. This specimen is missing the postzygapophyses save for a small portion on the right side, in addition to the posterior part of the centrum. The prezygapophyses are also damaged. This vertebra differs from the holotype mid-trunk vertebra (RRBP 10041) in exhibiting a neural spine that does not extend anteriorly past the posterior edge of the zygosphenal facet, a more depressed cotyle, and a relatively thick hemal keel.

#### Comparisons

These vertebrae are referable to Booidea on the basis of the restricted anteroposterior dimension combined with the large width [39], as well as overall robusticity, particularly of the zygosphene [14]. Two non-booid families (Madtsoiidae and Tropidophiidae) and one family of uncertain placement (Palaeophiidae) exhibit similar vertebral morphologies. However, *Rukwanyoka holmani* lacks the apomorphic pterapophyses of palaeophiids and large parazygantral foramina of madtsoiids. It is distinguishable from tropidophiids in having a relatively wide centrum along with the combination of an anteroposteriorly short centrum lacking a hypapophysis [10], [98].

Due to a high degree of morphological conservation, assignment to a booid family is difficult when working only with vertebral material [39], but *Rukwanyoka holmani* exhibits a number of diagnostic features. The relatively high neural spine and absence of hypapophyses in mid-trunk vertebrae suggests affinity with non-erycine Boidae or Pythonidae. Both families exhibit diversity in vertebral morphology and overlap considerably. The presence of paracotylar foramina is consistent with the condition observed in members of the Boinae [47] and *Candoia* [99], and has never been reported in pythonids, although this character is either homoplastic or plesiomorphic, appearing in both booid and non-booid families [100]. Bolyeriid snakes also exhibit paracotylar foramina, but *Rukwanyoka* lacks the mid-trunk hypapophyses present in bolyeriids [98]. The thin hemal keel in the mid-trunk is also characteristic of non-erycine boids. As in RRBP 10041, the neural spine of *B. constrictor* is thick posteriorly with an anterior blade that is notched in lateral view (e.g., YPM R 12323), although in *Boa* the neural spine is considerably taller than it is long. A similar condition occurs in *Epicrates cenchria*, *Corallus caninus*, and *Candoia aspera*; however, in each of these the thin anterior portion is smaller and tapers more strongly such that it is not a distinct blade. This condition is not present in *Calabaria* nor the Malagasy boids *Acrantophis dumerilii* and *Sanzinia madagascariensis*. The low zygosphene is considerably less robust than in some boid snakes, but its dorsoventral thickness is similar to that seen in smaller boids like *Candoia* and *Epicrates*.

Unknown Family.

Booid Morphotype B.

Figure 5A; Table 1.

**Figure 5:**
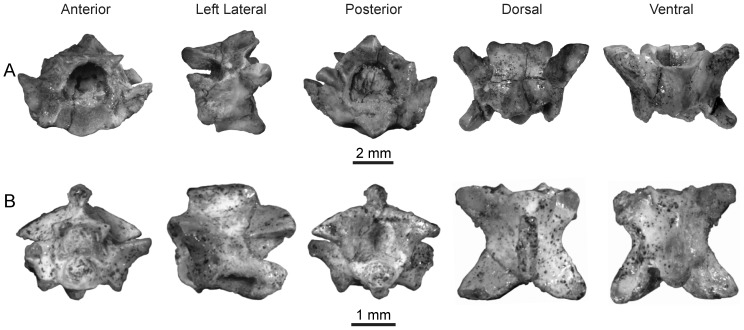
Indeterminate booid material from the late Oligocene of Tanzania. A, Booid Morphotype B (RRBP 11490) from the late Oligocene of Tanzania, posterior trunk vertebra; B, Booid Morphotype C (RRBP 04100) from the late Oligocene of Tanzania, mid-trunk vertebra.

#### Referred specimen

RRBP 11490, posterior trunk vertebra.

#### Locality

Late Oligocene Nsungwe Formation, locality Nsungwe2, Rukwa Rift Basin, southwestern Tanzania.

#### Description

The single specimen (RRBP 11490) is well preserved, deriving from the posterior trunk region, and missing only the neural spine, posterior portion of the neural arch, and the condyle. The vertebra is anteroposteriorly short and wide. It is also relatively large (largest available measurement: neural arch width at 4.3 mm) and second in size only to the holotype vertebra of *Rukwanyoka holmani*.

The neural arch is well preserved anteriorly, with damage only to the posterior portion overlying the zygantrum. Although the dorsal extent of the neural spine is not preserved, the base is restricted to the posterior half of the neural arch. The posterior portion is thick, and it tapers anteriorly so that it forms a triangle when seen in dorsal view, with a low ridge extending anteriorly to the zygosphene (Fig. 5A). The zygosphene is gracile and wider than the cotyle, with a flat superior surface and a straight anterior border with anteriorly projecting oval facets. Although the zygantrum is incomplete due to damage of the neural arch laminae, a single intrazygantral foramen is present bilaterally on the anterior wall of the zygantral fossa. The neural canal is large and wide.

Although abraded, the prezygapophyses preserve oval facets, with anterolaterally directed long axes. Prezygapophyses are inclined dorsolaterally at ∼ 68° from the median sagittal plane (Fig. 5A) and are missing accessory processes due to damage. Extending posteriorly from the prezygapophysis is a posterodorsally inclined interzygapophyseal ridge. This ridge is weakly concave laterally and terminates at the postzygapophysis. The left side of the specimen preserves a postzygapophysis with an oval facet.

The centrum is well preserved anteriorly except for the absence of synapophyseal facets. The condyle is not preserved. The cotyle is strongly depressed with notched ventrolateral margins. Lateral to the cotyle is a shallow and wide paracotylar fossa that lacks a paracotylar foramen. The ventral surface of the centrum bears a thick hemal keel that is unexpanded at the cotylar edge. Bilaterally there is a shallow subcentral fossa, bearing a small subcentral foramen. The lateral border of each subcentral fossa is formed by the weak subcentral ridge that extends posteromedially from the synapophysis.

#### Comparisons

RRBP 11490 exhibits morphological features typical of the posterior trunk, including a ventrolaterally notched cotyle, a relatively thick hemal keel, and a wide zygosphene with attenuated facets. Although RRBP 11490 is non-overlapping with *Rukwanyoka holmani*, differences in the aforementioned features are greater than would be expected within the column of a single species (based on observations of *Boa constrictor* YPM R 12323, *Python molurus* YPM R 12545). The neural spine is considerably shorter anteroposteriorly than that of *R. holmani*. Although anteroposterior restriction in the length of the neural spine can occur in posterior trunk vertebrae, this degree of difference would not be expected in a single taxon.

The specimen may be referred to Booidea on the basis of its great robusticity, and low anteroposterior length to mediolateral width. It cannot be more precisely placed, owing to the fragmentary nature and difficulty in comparing posterior trunk vertebrae to previously described mid-trunk vertebrae.

Booid Morphotype C.

Figure 5B; Table 1.

#### Referred specimen

RRBP 04100, mid-trunk vertebra.

#### Locality

Late Oligocene Nsungwe Formation, locality TZ-01, Rukwa Rift Basin, southwestern Tanzania.

#### Description

RRBP 04100 is about as wide as long, with a strong waisting in dorsal view. It is small, with the distance between pre- and postzygapophyses at 2.7 mm (centrum length uncertain due to incomplete preservation; Table 1).

The neural spine is short and anteroposteriorly elongate, with the anterior edge arising at the level of the zygosphenal facet. The neural spine is transversely thick and further expanded near its dorsal extremity so that it has a wide dorsal edge. There is a small anterior projection but no evidence of a posterior one. Neural arch laminae are gently convex dorsally with a strong posterior notch. The zygosphene is gracile, with convex dorsal and anterior edges. The zygantral facets do not project posteriorly. The neural canal is large and about as wide as it is tall.

The zygapophyses are nearly complete, missing only the lateral part of the left prezygapophysis. The facet of the right prezygapophysis is oval. The long axis of the facet is oriented anterolaterally, but closer to lateral than anterior. The prezygapophyseal accessory process is broken away, but the damaged base reveals it to be dorsoventrally flattened. The postzygapophyses are complete and have oval facets with the long axis oriented posterolaterally. The zygapophyses are dorsolaterally inclined at 75° from the median sagittal plane. The pre- and postzygapophyses are connected by a horizontal interzygapophyseal ridge.

On the centrum, the hemal keel and condyle are abraded, and neither synapophysis preserves a complete facet. The cotyle is wider than tall and deep. A shallow paracotylar fossa is located lateral to the cotyle and lacks foramina. The synapophyses are poorly preserved, but the parapophyseal portion projects ventrally below the cotyle, the former being well separated by a gap from the latter. The hemal keel is flattened and thick, expanding anteriorly. Although difficult to ascertain due to damage to the condyle, the keel appears to project strongly ventrally. Lateral to the hemal keel is a shallow subcentral fossa that bears a minute subcentral foramen. The subcentral ridges are low and do not extend all the way to the condyle.

#### Comparisons

RRBP 04100 preserves an unusual combination of features, including a low, anteroposteriorly-elongate and thick neural spine, a low centrum length to neural arch width ratio, and an extremely gracile zygosphene. The neural spine is reminiscent of “Boinae A” from the Ehrenstein 12 locality (Oligocene) in Germany [31]. However, the zygosphene of “Boinae A” is of a more typical thickness for booid snakes and is furthermore concave dorsally. *Bavarioboa ultima* from the Miocene of Germany also exhibits a low, long and thick neural spine, but again exhibits a more robust zygosphene that is concave dorsally [101]. The low and thick neural spine also suggests comparisons with erycine snakes [14], [39], [102], but in that clade the neural spine is typically restricted in anteroposterior dimensions. The genus *Falseryx* has a low neural spine and gracile zygosphene, but the neural spine is restricted to a position posterior to the zygosphenal facets [10]; the same is true of the tropidophiids *Platyspondylia* and *Rottophis* [18], [103]. Caenophidians often have low, anteroposteriorly elongate neural spines [14], [39], hence comparisons with those taxa are similarly unhelpful. Atractaspidines and aparallactines have more elongate vertebrae with thinner neural spines that are considerably lower than even RRBP 04100 [104]–[106]. The weight of the evidence supports placement of this specimen in Booidea, but additional material is needed to confirm this assignment.

Caenophidia Hoffstetter, 1939 [107].

Colubroidea Oppel, 1811 [92].

Colubroid Morphotype A.

Figure 6; Table 2.

**Figure 6:**
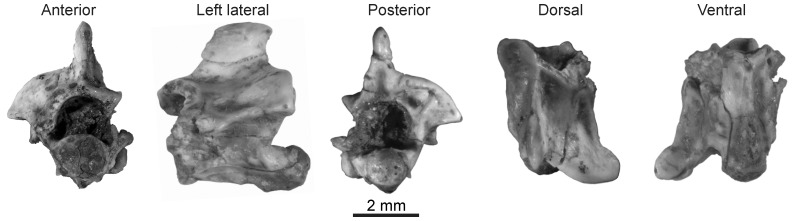
Anterior or mid-trunk vertebra of Colubroid Morphotype A (RRBP 07101) from the late Oligocene of Tanzania. Note that the lateral view is reversed.

**Table 2:** Measurements (in mm) of the colubroid vertebrae in the collection.

Taxon	Specimen	CL	NAW	ZSW	CTW	CTH	CNW	CNH	PRW	POW	PrPO	NH	HH
Colubroid Morphotype A	07101	–	–	–	1.7*	1.3	–	–	–	–	–	2.1	–
Colubroid Morphotype B	04154	3.4	2.6	–	–	1.2*	1.5	1.3	–	5.0	–	–	0.4
Colubroid Morphotype B	05306	–	2.5	1.8	–	–	1.2	1.1	–	4.1	–	–	0.2
Colubroid Morphotype B	06065	5.1	3.4	2.8	2.3	1.7	2.0	1.6	6.6	6.9	6.0	–	–
Colubroid Morphotype B	06013	–	2.6	2.0	–	1.1	–	–	–	–	–	–	–
Colubroid Morphotype B	06153	–	2.5	2.2	1.4	1.3	–	–	4.6	–	–	–	–
Colubroid Morphotype B	07115 (anterior)	–	2.5	2.2	1.5	1.3	–	–	5.4	–	4.0	–	–
Colubroid Morphotype B	07193	–	1.6	1.5	1.0	1.0	–	–	–	–	2.8	–	0.1
Colubroid Morphotype B	07437	3.7	2.4	2.4	1.6	1.2	1.4	1.1	4.7	4.9	4.0	1.3	0.9
Colubroid Morphotype B	09008	4.1*	2.9*	–	1.9	–	1.7	1.5	–	–	–	–	–
Colubroid Morphotype B	09016	3.5	2.3	1.8	1.5	1.3	–	1.1	4.6	–	4.2	–	0.2
Colubroid Morphotype B	09250	3.3	2.3	2.3	1.5	1.2	1.5	1.3	–	–	4.2	–	–
Colubroid Morphotype B	10045	–	2.6	2.5	1.6	1.2*	–	–	–	3.9	4.2	–	0.4
Colubroid Morphotype B	11350	–	2.0	1.6	1.0	0.9	–	–	–	3.5	3.0	–	–
Colubroid Morphotype C	07674	3.2*	2.7	1.6	1.4	1.3	–	–	3.5	3.2	3.8	0.7	–
Elapid Morphotype A	04320	3.1	2.1	1.9	1.2	1.0	–	0.9	–	–	3.3	0.9	0.6
Elapid Morphotype B	07257	4.5	4.3	3.1	2.4	2.0	2.0	1.9	–	–	–	–	0.9

**CL**, centrum length; **CNH**, condyle height; **CNW**, condyle width; **CTH**, cotyle height; **CTW**, cotyle width; **HH**, hypapophyseal height; **NAW**, neural arch width; **NH**; neural spine height; **POW**, width across the postzygapophyses; **PrPo**, distance from the anterior edge of the prezygapophysis to the posterior edge of the postzygapophysis; **PRW**, width across the prezygapophyses; **ZSW**, zygosphene width.

*slight underestimates of values due to erosion or breakage.

#### Referred specimen

RRBP 07101, anterior or mid-trunk vertebra.

#### Locality

Late Oligocene Nsungwe Formation, locality TZ-01, Rukwa Rift Basin, southwestern Tanzania.

#### Description

Colubroid Morphotype A is represented by a single fragmentary mid-trunk vertebra estimated at ∼ 4 mm in total length (Fig. 6; Table 2). The neural arch is tall with laterally convex laminae and a tall, thick neural spine that is posteriorly inclined such that it overhangs the zygantrum. The neural spine exhibits a convex dorsal margin and is anteroposteriorly short, situated entirely posterior to the zygosphene. Although half of the zygosphene is damaged, the intact half is clearly gracile and projects strongly anteriorly. The intact portion of the zygantrum preserves a facet that projects posteriorly beyond the edge of the neural arch. The neural canal is large and taller than wide. The right postzygapophysis is the only zygapophysis preserved and bears a small oval facet that is inclined dorsolaterally at 85° from the median sagittal plane. There is no epizygapophyseal spine or postzygapophyseal foramen. The interzygapophyseal ridge is preserved, well-marked with a posterodorsal inclination and a weak lateral concavity.

The centrum is damaged and preserves little intact morphology. The cotyle is depressed and deep, with a concave ventral border. Although minimally preserved, the condyle appears to have been upturned. The hemal keel is spatulate [97] with a thin anterior portion, but it is broken along the posterior half such that the extent of its ventral projection is unknown (e.g., whether or not it exhibited a prominent hypapophysis). The subcentral fossa preserved lateral to the hemal keel is shallow and appears to lack subcentral foramina. Subcentral ridges are distinct.

#### Comparisons

This vertebra is poorly preserved, making taxonomic comparisons difficult. It is clearly colubroid, as indicated by the elongate nature of the centrum, the gracile build of the zygosphene and the overall gracility of the vertebra. Poor preservation does not permit definitive serial assignment of the vertebra, because the hemal keel/hypapophysis is broken and the zygapophyses are poorly preserved. Although the great height of the neural spine is suggestive of an anterior trunk vertebra, the posterolateral orientation of the long axis of the postzygapophysis resembles a mid-trunk vertebra. As such, more complete examples of this morphotype are required to further refine the intracolumnar morphology of Colubroid Morphotype A.

There are very few Oligocene colubroid snakes with which to compare this material. All are very small, gracile specimens referred to Colubridae [28]–[30], [108]. Colubroid Morphotype A can be excluded from *Texasophis*, *Nebraskophis*, and *Floridaophis* on the basis of its tall neural spine and vaulted neural arch [28], [29]. Comparisons with *Natrix mlynarskii* are difficult due to the fragmentary nature of the material, but Colubroid Morphotype A has a more vaulted neural arch and a posteriorly inclined neural spine. It additionally lacks the parazygantral foramen and epizygapophyseal spine common in natricine snakes.

Colubroid Morphotype B.

Figure 7; Table 2.

**Figure 7:**
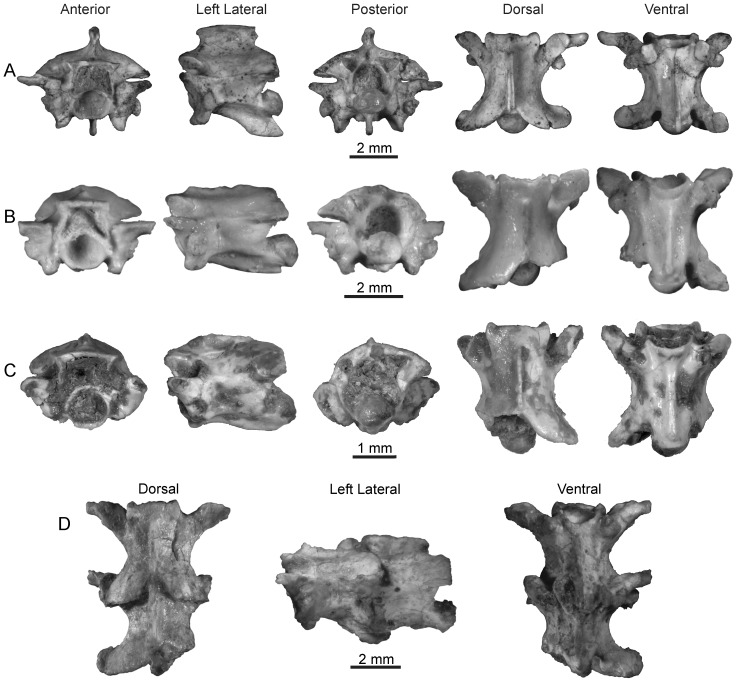
Colubroid Morphotype B from the late Oligocene of Tanzania. A, RRBP 07437, anterior trunk vertebra; B, RRBP 09016, mid-trunk vertebra; C, RRBP 07193, posterior trunk vertebra. Note that the lateral view is reversed; D, RRBP 07115, two pathologically fused anterior trunk vertebrae.

#### Referred specimens

RRBP 06065, RRBP 07115, RRBP 07437, RRBP 09008, RRBP 09250, anterior trunk vertebrae; RRBP 04154, RRBP 05306, RRBP 06013B, RRBP 09016, RRBP 10045, RRBP 11350, mid-trunk vertebrae; RRBP 06153, RRBP 07193, posterior trunk vertebrae.

#### Localities

Late Oligocene Nsungwe Formation, localities TZ-01, TZ-01S, TZP-2, and Nsungwe 2, Rukwa Rift Basin, southwestern Tanzania.

#### Description

Vertebrae of this morphotype make up the majority of snake specimens collected from the Nsungwe fauna and range in size from 2.8 to 6.7 mm in length (Table 2). There are specimens representing all regions of the vertebral column (Fig. 7), with most arising from the anterior and mid-trunk. Mid-trunk vertebral morphology is described as a baseline, and then variation due to regional differences is described.

The neural arch is low and has a convex border in posterior view (Fig. 7). The neural spine is incomplete on all mid-trunk vertebrae, but remnants indicate that it is thin at the base and anteroposteriorly elongate, arising at the level of the zygosphenal facets. The zygosphene is gracile, with a convex dorsal edge and a straight or weakly concave (e.g., RRBP 10045) anterior margin. The zygantrum is low and the dorsal portion of the neural canal separates the zygantral facets. It is unclear whether intrazygantral foramina are present. The neural canal is large, and wider than tall.

Among the various mid-trunk vertebrae collected, both pre- and postzygapophyses are represented by complete examples, but no prezygapophyseal accessory processes are preserved. The prezygapophyses bear an oval facet that ranges from relatively narrow to wide, likely dependent on serial position within the axial column. The facets are weakly inclined, ranging between 82.4° and 87.8° from the median sagittal plane. The postzygapophyseal facet is usually oval, although one specimen (RRBP 11350; Fig. 7B) exhibits a subrectangular facet. With the exception of RRBP 05306, a single, large postzygapophyseal foramen occurs dorsal to the facet. Only one mid-trunk vertebra (RRBP 11350) exhibits evidence of an epizygapophyseal spine in the form of an expansion immediately dorsal to the postzygapophysis (Fig. 7B), although the tip of this spine is broken away.

The centrum is elongate and lacks a hypapophysis. The cotyle is round and large. Lateral to the cotyle the paracotylar fossa is deep and contains a single paracotylar foramen situated close to cotylar mid-height. The hemal keel is gladiate (sensu [97]) and bounded laterally by a shallow subcentral fossa; the weak subcentral ridges compose the lateral border of the fossa. These are posteromedially directed and extend to the condyle. A subcentral foramen is present bilaterally and minute, except in RRBP 09016 where it is absent altogether.

Four typical and two pathologically fused vertebrae represent the anterior trunk region. In overall morphology these are similar to mid-trunk vertebrae. The neural spine is dorsoventrally short and anteroposteriorly long, reaching anteriorly to the level of the zygosphenal facet. The dorsal margin of the neural spine expands in the anteroposterior plane, resulting in a slight overhang anteriorly and posteriorly (Fig. 7A). RRBP 07115 preserves large epizygapophyseal spines that project laterally. The zygapophyses are more strongly inclined in the anterior trunk, ranging from 70° to 82° from the mid-sagittal plane. Three specimens (RRBP 06065, RRPB 07115, and RRBP 07347; Fig. 7A) preserve elongate, dorsoventrally compressed prezygapophyseal accessory processes with rounded tips. In RRBP 07347 the process projects laterally and exhibits a weak anterior curvature, but this curvature is absent in the other two specimens. The morphology of the synapophyses is preserved completely in RRBP 07347 and RRBP 06065; the diapophysis is strongly convex and laterally prominent, and well demarcated from the convex parapophysis. There is also a weak constriction at the junction of these two portions of the synapophysis (Fig. 7A). The short hypapophysis exhibits a sinuous anterior border, a pointed tip, and angles posteriorly, but does not project beyond the condyle in RRBP 07437. Although obscured by pathology, the hypapophysis is shorter in RRBP 07115 (Fig. 7D), suggesting a position very near the anterior to mid-trunk transition. Prominent parapophyseal processes equal in length to the parapophyseal facet are preserved on RRBP 06065, RRBP 07115, RRBP 07437 and RRBP 09250 (Fig. 7A).

One vertebra (RRBP 07193; Fig. 7C) represents the posterior trunk region, and one mid-trunk vertebra (RRBP 09016) arises from a posterior region of the mid-trunk near the transition zone. The posterior trunk vertebra is similar to the mid-trunk series. Postzygapophyseal foramina are not present, but this may be due to the loss of the lateral portion of the postzygapophyseal process. The vertebra also differs in having deeper subcentral fossae that open in anterior view between the remnants of the synapophyses and the cotyle, weakly incising the ventrolateral edge of the latter. The posterior trunk vertebra also differs in having a relatively thicker hemal keel. The mid-trunk vertebra from the posterior portion of that region exhibits signs of these morphological changes, but they are only incipiently developed.

#### Comparisons

This taxon differs from Colubroid Morphotype A (Fig. 6) in exhibiting an anteroposteriorly elongate and low neural spine with anterior and posterior dorsal processes, a low neural arch, a neural canal that is wider than tall, and a gladiate rather than spatulate hemal keel.

With the exceptions noted below, vertebral morphology in the colubroid families and subfamilies is poorly characterized. Although a large number of snakes have previously been united under the umbrella of Colubridae, the actual relationships among them are still being resolved. This renders comparisons of colubroid vertebral morphology difficult. Moreover, some vertebral features likely represent homoplasies, occurring in multiple clades. The absence of posterior trunk hypapophyses and relatively small cotyle-condyle complex indicate that Colubroid Morphotype B is not a viperid [14], [39]. This morphotype is also excluded from Xenodermatidae, because that clade exhibits vertebrae marked by apomorphic accessory processes off both zygapophyses and lateral expansions of the neural spine [109]. Moreover, the absence of hypapophyses in the posterior part of the trunk suggests against referral to Elapidae, Lamprophiidae (excluding Psammophiinae), Natricinae, or Pseudoxyrhophiinae [14], [39], [110], [111]. However, the actual value of that character bears closer examination, as shown by an absence of posterior trunk hypapophyses in at least one natricine snake [112], [113] and in the great reduction of these processes to a low knob in the pseudoxyrhophiine *Duberria lutrix* (pers obs; USNM 145120, USNM 145121). The height of the neural spine and the elongate nature of the prezygapophyseal accessory processes also suggest against assignment to Elapidae. Unfortunately, psammophiine and colubrine snakes cannot be differentiated based on vertebral morphology at this time.

The three Oligocene North American colubroid genera *Texasophis*, *Nebraskophis*, and *Floridaophis*, are small snakes with depressed neural arches and short neural spines [21], [28], [114]; *Texasophis* and *Nebraskophis* additionally have much shorter prezygapophyseal accessory processes [28], [114], and *Floridaophis* has narrow prezygapophyseal accessory processes which are acuminate [97] rather than terminating bluntly [28]. They are more gracile, and with the exception of *Texasophis galbreathi*, feature a flatter hemal keel [28], [29], [114]. A fourth, unnamed, North American colubrid differs from Colubroid Morphotype B in having a thicker, less defined hemal keel, an anteroposteriorly restricted neural spine, and a straight anterior border to the zygosphene [21]. The European natricine *Natrix mlynarskii* differs from Colubroid Morphotype B in having hypapophyses in the posterior trunk, parazygantral foramina, and in having a triangular, protruding keel below the cotyle.

There is a great range in size of material attributed to this morphotype. This is at least partly a result of intracolumnar variation in size, and the smallest vertebrae in this collection are from the precloacal (sensu [96]) and caudal regions. However, some of this no doubt relates to intraspecific variation, with the largest vertebra (RRBP 06065) representing an anterior trunk vertebra considerably larger (6.7 mm centrum length) than the other anterior trunk vertebrae recovered (Table 1). Nevertheless, even this was not a large snake, and comparison with extant material suggests an estimated size of 1 m.

Colubroid Morphotype C.

Figure 8a; Table 2.

**Figure 8:**
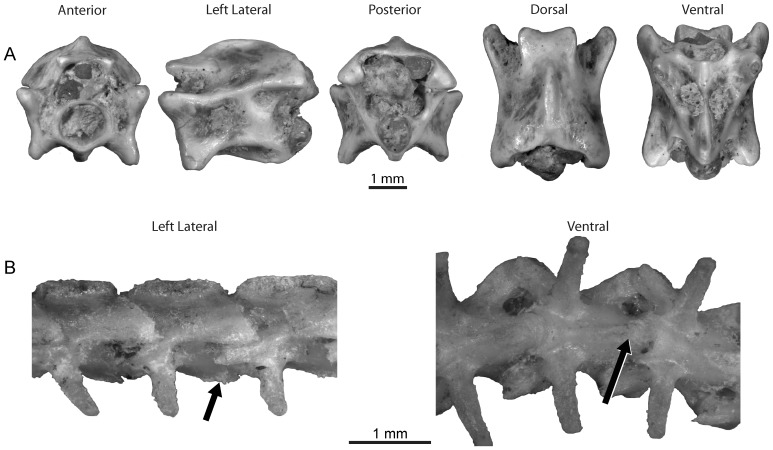
Caudal vertebrae of Colubroid Morphotype C (RRBP 07674) from the late Oligocene of Tanzania with comparison to extant *Duberria lutrix*. A, RRBP 07674, caudal vertebra; B, *Duberria lutrix* (CM 145121), caudal series.

#### Referred specimen

RRBP 07674, caudal vertebra.

#### Locality

Late Oligocene Nsungwe Formation, locality TZ-01, Rukwa Rift Basin, southwestern Tanzania.

#### Description

This morphotype is represented by a single caudal vertebra preserving an unusual morphology. The vertebra is gently waisted, with a depressed neural arch and narrow but distinct zygosphene. Only the base of the pleurapophysis is preserved, and rather than paired hemapophyses, the specimen apparently had only a single midline process of uncertain length.

The neural arch is low, with a convex dorsal border. The neural spine is incompletely preserved, and lies entirely posterior to the zygosphene. Due to erosion the neural spine is rendered as a low, rounded ridge, but its specific morphology is impossible to ascertain. The zygosphene is gracile and narrow, with a flat dorsal edge and a concave anterior edge. The facets are distinct and oval. The neural canal is relatively wide, exceeding the width of the cotyle.

The zygapophyses are completely preserved. The prezygapophyseal facet is a narrow oval with a long axis oriented anterolaterally (but more anteriorly than laterally). The facet is inclined dorsolaterally at ∼75° from the median sagittal plane. Projecting anteriorly from the facet is a thick, rounded prezygapophyseal accessory process. This process is poorly defined, but it is not directed parallel to the facet. The postzygapophysis also bears a narrow oval facet, with the long axis of the facet directed almost directly posteriorly. There is no postzygapophyseal foramen. The interzygapophyseal ridge is weakly inclined posterodorsally, and low but well defined.

In ventral view, the centrum has a strong posterior taper giving it a triangular appearance. The cotyle is as wide as tall, and ventrally tapers to a point at the midline. The condyle is poorly preserved, but it is not upturned. The pleurapophyses are unpreserved. There is a lateral foramen in a shallow fossa posterodorsal to the base of the left pleurapophysis. Extending posteriorly from the pleurapophysis is a well-defined subcentral ridge that is angled posteromedially. A single midline ridge is present on the ventral surface of the centrum. This ridge is low, rounded and narrowest just anterior to the midpoint. The posterior extremity of the ridge is damaged such that it is not possible to ascertain whether it is a hypapophysis or just a deeper portion of the hemal keel. There is no evidence for paired hemapophyses. Subcentral lymphatic fossae are present and shallow.

#### Comparisons

Postcloacal vertebrae differ considerably in morphology from the trunk, and as such are difficult to properly attribute to a particular morphotype. This difficulty is exacerbated in comparisons with Colubroid Morphotype A, represented by only a single vertebra, and it is possible that both are derived from the same species. It is also possible that the caudal vertebra Colubroid Morphotype C pertains to Colubroid Morphotype B; however, the extreme reduction in the neural spine argues against this. Two other caudal vertebrae (RRBP 04153 and RRBP 09284; see below) are present in the collection, and they partly preserve typical paired hemapophyses. The better-preserved specimen (RRBP 09284) has a higher, more anteroposteriorly restricted neural spine and is a better candidate for attribution to Colubroid Morphotype B.

Caudal vertebra RRBP 07674 is notable in displaying an unusual combination of features, including typical caudal features like pleurapophyses, a narrow zygosphene with nearly vertical facets, narrow zygapophyses with their long axes rotated toward the midline, and anteriorly directed prezygapophyseal accessory processes. The presence of a single hemal keel or hypapophysis instead of paired hemapophyses is unusual. Some basal snakes lack ventral projections (scolecophidians [115], *Anilius* [14], and *Cylindrophis* [14], [115]), and at least one tropidophiid bears hemapophyses on a common midline process (*Tropidophis haetianus*; seen in YPM R 13579). The elongate nature of the vertebra indicates it belongs to a colubroid, a clade in which hemapophyses are nearly universally present. The pseudoxyrhophiine snake *Duberria lutrix* lacks hemapophyses, and instead exhibits a strongly developed, thick hemal keel that does not project strongly enough to be considered a hypapophysis (e.g., specimens CM 145120, CM 145121; Fig. 8B). The distribution of caudal hypapophyses is uncertain, as other pseudoxyrhophiines have typical caudal hemapophyses; it is possible that it is an autapomorphy of *Duberria*, or that it is autapomorphic for the African pseudoxyrhophiine subclade including *Duberria*, or that it is distributed homoplastically in *Duberria* and RRBP 07674. *Duberria* differs from Colubroid Morphotype C in having pleurapophyses that project laterally throughout the tail; although these processes are damaged in Colubroid Morphotype C, the broken base faces nearly ventrally indicating they were not similarly oriented. In addition, the subcentral ridges are less developed and the prezygapophyseal accessory process is better defined and more pointed in *Duberria*.

There is often a short anterior caudal region where the cloacal hypapophyses are gradually replaced by paired hemapophyses [116]. One or two vertebrae in this region may therefore bear a single midline process. However, anterior caudal vertebrae typically preserve laterally directed prezygapophyseal accessory processes and the zygapophyseal facets are typically not parallel to the main vertebral axis as they are in RRBP 07674.

Colubroidea Oppel, 1811 [92].

Elapidae Boie, 1827 [117].

Elapid Morphotype A.

Figure 9A; Table 2.

**Figure 9:**
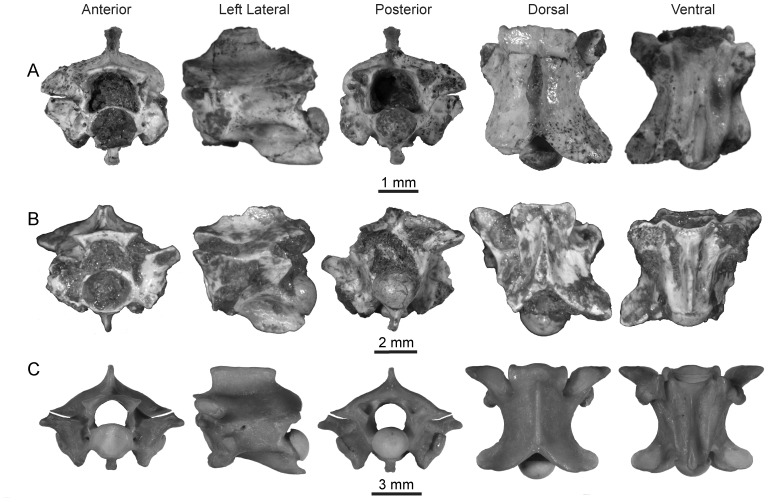
Elapid snakes from the late Oligocene of Tanzania with comparison to extant *Naja nigricollis*. A, Elapid Morphotype A (RRBP 04320), posterior trunk vertebra. Note the lateral view is reversed; B, Elapid Morphotype B (RRBP 07257), posterior trunk vertebra; C, *Naja nigricollis* (USNM 320723), posterior trunk vertebra.

#### Referred specimen

RRBP 04320, posterior trunk vertebra.

#### Locality

Late Oligocene Nsungwe Formation, locality TZ-01, Rukwa Rift Basin, southwestern Tanzania.

#### Description

This morphotype is represented by a single, strongly waisted posterior trunk vertebra that retains a well-developed hypapophysis. Its position as a posterior trunk vertebra is indicated by the presence of deep subcentral lymphatic fossae that are visibly open in anterior view and that weakly incise the cotyle.

The neural arch is low, with a convex dorsal border in anterior view. The neural spine is incomplete, but its base extends anteriorly to the zygosphene. Its anterior border slopes posteriorly toward a flattened dorsal edge in lateral view. The superior portion is expanded laterally. The zygosphene is gracile, with a dorsal convexity. Its anterior border is also weakly convex. The zygantrum is dorsoventrally short, with lateral triangular fossae separated by the dorsal extent of the neural canal.

Both right zygapophyses are partially preserved with little remaining of the left articular processes. The prezygapophysis lacks its distal extent including the accessory process, but enough remains of the facet to see that it is oval with its long axis directed anterolaterally. The right postzygapophyseal facet is more nearly orbicular than the prezygapophyseal facet. The zygapophyses are inclined dorsolaterally to about 80° from the mid-sagittal plane. Postzygapophyseal foramina are lacking. The interzygapophyseal ridge is straight in lateral view with a sharp peak over the posterior half of its length.

The centrum is elongate with straight sides in ventral view. The cotyle is circular, with weak notches along the ventrolateral aspect corresponding to the anterior opening of the subcentral lymphatic fossae. Lateral to the cotyle the paracotylar fossa is shallow and wide and bears a single large foramen at the dorsoventral midpoint of the cotyle. Only the right half of the condyle is preserved, and it is weakly upturned with a distinct lip separating the condyle from the centrum (Fig. 9A). There is a weak precondylar constriction. The synapophysis is eroded, and the condition of the parapophyseal process cannot be assessed. Bilaterally, a lateral foramen is present in a deep fossa dorsolateral to the synapophysis. The hypapophysis is thick and short, not projecting posteriorly past the condyle. It arises from the posterior half of the centrum, with a sinuous anterior border and a pointed distal tip. The ventrally-facing edge is transversely expanded. A low keel extends anteriorly from the hypapophysis up to the cotyle, where it is expanded and bears the ventrolateral cotylar processes. Lateral to the hypapophysis is the deep and narrow subcentral lymphatic fossa, bounded laterally by a prominent, rounded subcentral ridge. This ridge extends posteriorly to the notch that forms the intervertebral foramen. Bilaterally, a single subcentral foramen is set at the anterior edge of the hypapophysis in the subcentral fossa.

#### Comparisons

This specimen is morphologically distinct from all other Nsungwe Formation colubroid morphotypes in preserving: a lateral expansion of the neural spine and hypapophysis at their distal edges not observed in either Colubroid Morphotype A or Colubroid Morphotype B; a relatively shorter neural spine than observed in Colubroid Morphotype A; presence of a hypapophysis on posterior trunk vertebrae not observed in Colubroid Morphotype B; presence of ventrolateral cotylar processes not observed in posterior trunk vertebrae of Colubroid Morphotype B.

Elapid Morphotype A shares the following features with Elapidae: a low, recurved hypapophysis persistent in the posterior trunk region [39], [107], [113]; the absence of a postzygapophyseal foramen (note that this is also occasionally absent in Colubrinae, but that colubrines never have posterior trunk hypapophyses). Features consistent with terrestrial elapids but not exclusive to the clade include a low, anteroposteriorly elongate neural spine and well-developed subcentral ridges (also present in some colubrids and lamprophiids). Although very little of the synapophyses is preserved, enough of the left side is preserved to indicate that if a parapophyseal process was present, it was reduced as it is in Elapidae [107]. The short, thick hypapophysis preserving a flattened ventral edge is similar to the condition observed in posterior trunk vertebrae of *Naja*, a taxon with individuals that also exhibit a lateral expansion of hypapophyses at its ventral limit (seen in *Naja haje* CM 145401 and *Naja nigricollis* USNM 320723; see Fig. 9C).

As with the previous morphotypes, brief comparisons with other Oligocene snakes reveals this is a new taxon. The neural spine is too tall for referral to the North American colubrids; these species are also more gracile. Elapid Morphotype A differs from *Natrix mlynarskii* in lacking parazygantral foramina, in having a more gracile zygosphene, in being relatively shorter anteroposteriorly, and in lacking the large expansion of the keel ventral to the cotyle. Elapid Morphotype A does share some features with the posterior trunk vertebrae of the Miocene natricine *Natrix sansaniensis* from Europe [118]. In particular, the shape of the hypapophysis is very similar in being dorsoventrally short and curved with a posteriorly-directed point [11], [38]. However, the hypapophysis in Elapid Morphotype A has a nearly horizontal edge, rather than being posteroventrally inclined. The centrum is also anteroposteriorly shorter than that of *N. sansaniensis*, and the neural arch exhibits weaker convexity of the lamina.

Elapid Morphotype B.

Figure 9B; Table 2.

#### Referred specimen

RRBP 07257, posterior trunk vertebra.

#### Locality

Late Oligocene Nsungwe Formation, locality TZ-01S, Rukwa Rift Basin, southwestern Tanzania.

#### Description

The only vertebra referred to this morphotype is a large posterior trunk vertebra that bears a plate-like hypapophysis. In overall aspect, it is wider than long, with a gracile zygosphene and depressed neural arch. The neural spine and synapophyses are damaged, as is one each of the pre- and postzygapophyses.

Although the neural spine is damaged, the well-preserved base indicates it was anteroposteriorly elongate, reaching the level of the midpoint of the zygosphenal facet. It is widest posteriorly and tapers anteriorly. The gracile zygosphene exhibits a weakly concave anterior edge and a sinuous dorsal margin. It bears elongate oval facets. The zygantrum is damaged, but what remains reveals it to be dorsoventrally short. The dorsal extent of the neural canal is at the same level as the midpoint of the zygantral facet. This facet does not project posteriorly. The right neural arch lamina is completely preserved and is straight. A strong notch is present on the posterior edge of the neural arch. The neural canal is wider than tall.

One of each zygapophyseal pair is preserved. The left prezygapophysis bears an oval facet, the long axis of which is oriented anterolaterally. It is strongly inclined dorsolaterally at ∼ 74° from the mid-sagittal plane. The prezygapophyseal accessory process is not preserved. The right postzygapophysis bears an oval facet, the long axis of which is oriented posterolaterally. It is also inclined dorsolaterally at ∼ 69° relative to the mid-sagittal plane. There is neither a postzygapophyseal foramen nor an epizygapophyseal spine. A distinct, posterodorsally inclined interzygapophyseal ridge is positioned between the zygapophyses.

The cotyle and condyle are completely preserved. They are large and round, and the cotyle is weakly notched ventrolaterally by the subcentral fossae. Lateral to the cotyle, the paracotylar fossa is deep; the presence of paracotylar foramina is uncertain. The condyle is weakly upturned. The opening of the subcentral lymphatic fossa separates the cotyle from the synapophysis. Damage prevents any further description of the synapophysis. The thick hypapophysis is plate-like, with a convex ventral edge. Although it is posteroventrally inclined, it does not project posterior to the condyle. Lateral to the hypapophysis is a deep and narrow subcentral lymphatic fossa. Extending anteriorly from the hypapophysis to the cotyle is a peaked midline ridge that expands immediately posterior to the cotyle. The subcentral ridges are distinct and thick and extend posteriorly to the condyle.

#### Comparisons

Elapid Morphotype B shares with elapid snakes a low, posteriorly inclined, thick hypapophysis in posterior trunk vertebrae [39], [107], [113], [119], and with African elapids the absence of a postzygapophyseal foramen. Although RRBP 07257 derives from a more anterior part of the posterior trunk, it is distinguished from Elapid Morphotype A in having an anteriorly tapered neural spine and a relatively thinner hypapophysis, and in the absence of a precondylar constriction observed in Elapid Morphotype A.

### Indeterminate Specimens

RRBP 06119 (recovered from locality TZ-01; measurements in Table 3) is badly weathered with a high vertebral width to length ratio that only permits its referral to basal Alethinophidia. Two specimens pertain to booid snakes (Table 3); the first (RRBP 06179, recovered from locality TZ-01S) consists of only a centrum and partial neural arch, similar to *Rukwanyoka* in having a depressed cotyle and thickened hemal keel, but it is too damaged for a positive attribution; the second (RRBP 04332, recovered from locality TZ-01), is an anterior trunk vertebra from within the first ten postaxial vertebrae. Its morphology is typical for a vertebra of this region, and the lack of overlapping material makes positive attribution impossible. Three specimens are unidentified colubroids (measurements in Table 3). RRBP 04362 (recovered from locality TZ-01) is a weathered mid-trunk vertebra. RRBP 06067 (recovered from locality TZ-01S) is a weathered posterior trunk vertebra possibly pertaining to one of the elapid morphotypes. RRBP 07657 (recovered from locality Nsungwe 2 Big Wall) is the partial anterior face of a possible juvenile colubroid vertebra. Three others are well preserved colubroid vertebrae but cannot be positively attributed to a morphotype due to the high degree of intracolumnar variation in the regions from which they arise (Table 3). The first (RRBP 11381, recovered from locality TZ-01S) is from the precloacal region (sensu [96]) and exhibits features typical of the region. The other two (RRBP 04153, recovered from locality TZP-2, and RRBP 09284, recovered from locality Nsungwe 2) are caudal vertebrae that are distinct from Colubroid Morphotype C in having hemapophyses.

**Table 3:** Measurements (in mm) of the indeterminate vertebrae in the collection.

Taxon	Specimen	CL	NAW	ZSW	CTW	CTH	CNW	CNH	PRW	POW	PrPo	NH	HH
booid indet	04100	–	1.7	1.3	0.9	0.8	–	–	2.9	2.6	0.8	–	0.8
booid indet	04332	–	2.3	1.9	1.3	1.0	–	–	3.0	2.5	–	0.7	–
booid indet	06179	2.8*	3.6	–	2.0	1.5	–	–	–	–	3.4	–	–
colubroid indet	04153	3.4	2.5	–	1.6	1.2	1.3	–	–	–	4.2	–	0.1
colubroid indet	04362	–	1.9	–	1.1	1.0	–	–	–	–	–	–	0.1
colubroid indet	06067	3.5	2.6	–	1.4	1.3	1.2	1.2	–	–	–	–	–
colubroid indet	07657	–	–	1.8	1.2	1.1	–	–	–	–	–	–	–
colubroid indet	09284	2.5	1.9	1.2	0.8	0.9	0.7	0.7	–	2.7	3.1	–	–
colubroid indet	11381	–	1.6	1.4	0.7	0.7	–	–	–	–	–	–	–

**CL**, centrum length; **CNH**, condyle height; **CTH**, cotyle height; **CTW**, cotyle width; **HH**, hypapophyseal height; **NAW**, neural arch width; **NH**; neural spine height; **POW**, width across the postzygapophyses; **PrPo**, distance from the anterior edge of the prezygapophysis to the posterior edge of the postzygapophysis; **PRW**, width across the prezygapophyses; **ZSW**, zygosphene width.

*slight underestimates of values due to erosion or breakage.

## Discussion

### The Evolution of African Faunas

The Nsungwe Formation snake assemblage represents an important snapshot of Paleogene terrestrial snake evolution from southern Africa, and its age provides a critical window into the evolution of African snakes. The colubroid-dominated Nsungwe Formation predates the faunal shift that has been documented in the Miocene on northern continents [1]–[3], and is contemporaneous with the “dark period” for snakes in Europe [9], [10]. During this time, the relative abundance of snakes in vertebrate faunas from western and central Europe was dramatically lower than that observed in earlier and later faunas. Furthermore, the few remaining snakes tended to be small and presumably fossorial on the basis of vertebral morphology [9].

The Nsungwe Formation fauna provides critical new information to test whether the patterns observed in Europe and North America denote a more global phenomenon. Some similarities are apparent. For example, the Nsungwe snakes are all small; comparisons with vertebral specimens of extant snakes suggests that none of them exceeded 1 m in total length. As is the case in the Oligocene of Europe and North America, the snake diversity in the Nsungwe Formation is also relatively low, although this may be a function of a limited sample size recovered to date. Alternatively, the relative paucity of snake remains may actually reflect low ophidian diversity, particularly given that snakes represent only 30 of the 4000+ specimens collected from the Nsungwe Formation to date. But one surprising and important difference with European snake assemblages is the dominance of colubroid remains at Nsungwe, comprising more than 75% of the recovered material and five of the eight recognizable morphotypes. This is in strong contrast with contemporaneous faunas of Europe and North America, suggesting a more complex interplay of faunal turnover than has previously been recognized.

The faunal turnover that occurred on northern continents during the Miocene coincided with increased aridity and the spread of grassland environments [120]. Colubroid dominance in the Nsungwe Formation may have resulted from more open habitats resulting from seasonally dry conditions in the region during the late Oligocene [72], [121], providing an independent case supporting Savitzky’s [4] hypothesis that snake foraging mode evolution tracks environmental conditions. A paleoenvironmental reconstruction of seasonally dry habitats for the Nsungwe Formation is supported by sedimentological data [72] and is consistent with the more crestiform and mesiodistally elongate cheek tooth morphology exhibited by Nsungwe Formation micromammals relative to the conditions observed in earlier Paleogene faunas further north [122]. The broader significance of a colubroid-dominated fauna in the Nsungwe Formation is unclear, mostly owing to the very poor contemporaneous record from Africa. This fauna may be an isolated instance where conditions favored colubroid snakes over booids, implying a patchwork replacement pattern in Africa. Alternatively, fossils from the Nsungwe Formation may represent an accurate snapshot of the African continental ophidian fauna in the Late Oligocene, indicating a major departure from the pattern observed in Europe and North America. The collection of material from different localities sampling this critical time period is necessary in order to distinguish between these possibilities, but in either case the pattern of faunal overturn in Africa is different and/or more complex than that documented for northern continents.

The Nsungwe fauna additionally provides the oldest definitive boid from Africa in *Rukwanyoka holmani*. The only potentially older report is a single vertebra from the late Paleocene of Morocco, but that specimen is poorly preserved and was only tentatively referred to Boidae [45]. Erycine boids have been reported from the Oligocene of what is now the Arabian Peninsula [123], and today the family is represented by erycines and the enigmatic genus *Calabaria*. *Rukwanyoka* represents the first non-erycine boid known from eastern Africa, and provides a possible geographic connection between the West African *Calabaria* and the Malagasy boids.

### The Rise of Colubroid Dominance

Colubroid snakes may have been present in North Africa as early as the Late Cretaceous [27], but see [23], and they were present in southern Africa by the Eocene [22]. The early occurrence of colubroid snakes in Africa prompted Rage and colleagues [22] to suggest that the clade could have evolved in Africa; however, molecular evidence generally supports an Asian origin [86], [124], and the contemporaneous or older occurrence of colubroids in other continents [23], [25], [125] weakens support for an African origin for the clade. However, a large clade of mostly African colubrids likely did originate in Africa, possibly as long ago as the Late Eocene [37], [126], and the Colubroid Morphotypes A, B, and C may pertain to this radiation.

The presence of elapids in Africa at this time is somewhat unexpected. Although the larger clade evolved in Africa in the Eocene, the family Elapidae is thought to have appeared during the Oligocene in Asia, between 30–35 Ma [32], [37]. If elapid snakes are present in Africa at 24 Ma, elapids either rapidly dispersed back into Africa (but not Europe) before the latest Oligocene, or they evolved in Africa rather than in Asia, dispersing to reach Australasia by the beginning of the Miocene [40].

The faunal turnover in Europe and North America is generally attributed to a late Early Miocene invasion of colubroids ultimately originating in Asia [3], [6]. According to molecular divergence data, Asian colubrids joined the African snake fauna in the Late Oligocene or Early Miocene [36], [127]. The Nsungwe Formation fauna either predates or is contemporaneous with these movements, and therefore may capture endemic components of the southern African snake fauna of the middle Cenozoic. If Colubroid Morphotypes A, B or C can be definitively allied to the African radiation, the Nsungwe fauna presents early dominance of caenophidian snakes in Africa due to an autochthonous radiation; if they are instead allied to Colubridae, it indicates an earlier invasion and rise to dominance in Africa by that clade relative to these events in Europe and North America.

## Conclusions

Here we describe the oldest snake material recovered from eastern Africa, represented by at least eight different morphotypes. Significantly, this includes the oldest definitive booids and the first occurrence of elapids from continental Africa. All of the snakes described herein are small, with vertebrae ranging in length from 2.6 to just over 5 mm. Colubroid material dominates the collection, representing an unusual pattern for Paleogene faunas. In addition the number of colubroid morphotypes outnumbers that of booids (five to three). This implies an earlier start to the faunal overturn that resulted in modern snake assemblages, likely due to changing environmental conditions that favored active rather than ambush predators. Whether this reflects general patterns across Africa or is simply a local phenomenon can only be clarified by further work at contemporaneous localities. Rukwa Rift Basin localities are beginning to provide new data on the evolutionary diversification of Cenozoic African snakes, providing insights into paleoenvironmental shifts and how animals respond to environmental change through time.

## Supporting Information

Supporting Information S1 **Taxon and accession information for comparative materials used in study.**
(DOCX)

Supporting Information S2 **3-Dimensional PDF of Type specimen of** ***Rukwanyoka holmani*** **(RRBP 10041, mid-trunk vertebra) from the late Oligocene Nsungwe Formation of Tanzania.**
(PDF)
