# Supplementary material for: The Earliest Colubroid-Dominated Snake Fauna from Africa: Perspectives from the Late Oligocene Nsungwe Formation of Southwestern Tanzania
Source: PLoS One. 2014 Mar 19;9(3):e90415. doi: 10.1371/journal.pone.0090415 (PMC3960104; doi:10.1371/journal.pone.0090415)
Supplement: Supporting Information S1 — Taxon and accession information for comparative materials used in study. (DOCX) [file pone.0090415.s001.docx]

**Supporting Information: PLoS ONE**

The earliest colubroid-dominated snake fauna from Africa: Perspectives from the late Oligocene Nsungwe Formation of southwestern Tanzania.

Jacob A. McCartney^1,2,3^, Nancy J. Stevens^*,2,3^, Patrick M. O’Connor^2,3^

**^1^**Department of Anatomical Sciences, Health Sciences Center, Stony Brook University, Stony Brook, NY 11794-8081, U.S.A.,

**^2^** Department of Biomedical Sciences, 228 Irvine Hall, Heritage College of Osteopathic Medicine, Ohio University, Athens, Ohio 45701, U.S.A.,

^3^ Center for Ecology and Evolutionary Studies, Irvine Hall, Ohio University, Athens, Ohio 45701, U.S.A.

Taxon and accession information for comparative materials used in study.

Atractaspidinae: *Atractaspis irregularis* (USNM 297313)

Boidae: *Acrantophis dumerilii* (YPM R 11921); *Boa constrictor* (YPM R 12323); *Candoia aspera* (YPM R 15187); *Candoia bibroni* (MCZ 14516, YPM R 13884); *Candoia carinata* (USNM 348503, YPM R 13199); *Calabaria reinhardtii* (YPM R 11098); *Casarea dussumieri* (MCZ 49135); *Charina bottae* (YPM R 10608); *Corallus caninus* (MCZ 128139, YPM R 10816, YPM R 14980); *Epicrates cenchria* (YPM R 13810); *Epicrates inornatus* (MCZ 4648); *Eryx colubrinus* (MCZ 18380, MCZ 40304); *Eryx johnii* (YPM R 18263); *Eunectes murinus* (YPM R10657); *Lichanura roseofusca* (MCZ 8966); *Sanzinia madagascariensis* (YPM R 12241)

Colubridae: *Dasypeltis medici* (CM 145095); *Dasypeltis scabra* (CM 35891); *Dasypeltis scabra atra* (CM 145097, CM 145100); *Grayia smythii* (USNM 320720); *Natriciteres olivacea* (MCZ 131463, USNM 297314, YPM R 13169, YPM R 13171) *Philothamnus battersbyi* (CM 145224, CM 145225); *Scaphiophis albopunctatus* (MCZ 23068); *Spalerosophis diadema* (YPM R 13383); *Telescopus* sp. (CM 145744)

Elapidae: *Acanthophis antarcticus* (YPM R 13646); *Bungarus candidus* (MCZ 8015); *Dendroaspis angusticeps* (CM 145392); *Dendroaspis jamesoni* (YPM R 13380); *Dendroaspis polylepis* (CM 145393); *Dendroaspis viridis* (USNM 94445); *Hydrophis fasciatus* (YPM R 10681); *Lapemis hardwickii* (MCZ 12540); *Naja haje* (CM 114441, CM 145401); *Naja mossambica* (CM 145405); *Naja naja* (CM 37154; CM 114440); *Naja nigricollis* (USNM 320723); *Ophiophagus hannah* (MCZ 67054)

Lamprophiinae: *Lamprophis fuliginosis* (CM 114368; USNM 348620, YPM R 17193); *Lycodonomorphus bicolor* (MCZ 54901); *Lycophidion capense* (AMNH R 5259, CM 145195; CM 145196; USNM 142081)

Psammophiinae: *Malpolon monspessulanus* (AMNH R 73350, CM 145197); *Psammophis lineolatus* (USNM 14334); *Psammophis sibilans* (CM 145232; CM 145233); *Rhamphiophis oxyrhynchus* (USNM 320721)

Pseudoxyrhophiinae: *Duberria lutrix* (CM 145120, CM 145121); *Geodispsas laphystia* (MCZ 181165); *Liopholidophis dolicocercus* (MCZ 180409); *Leioheterodon geayi* (YPM R 13207); *Madagascarophis colubrinus* (USNM 149357)

Tropidophiidae: *Tropidophis haetianus* (YPM R 13579)
