# Supplementary figures and images for: The Earliest Colubroid-Dominated Snake Fauna from Africa: Perspectives from the Late Oligocene Nsungwe Formation of Southwestern Tanzania
Source: PLoS One. 2014 Mar 19;9(3):e90415. doi: 10.1371/journal.pone.0090415 (PMC3960104; doi:10.1371/journal.pone.0090415)

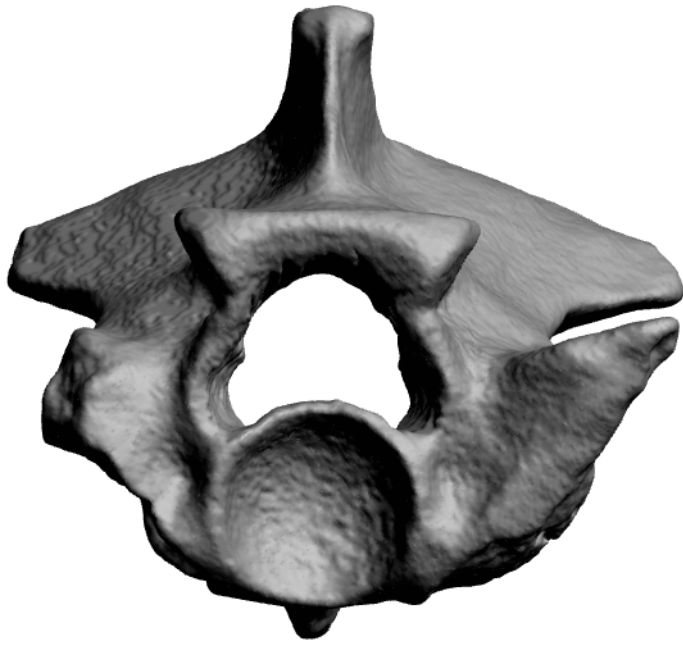

RRBP 10041

Supplement: Supporting Information S2 — 3-Dimensional PDF of Type specimen of Rukwanyoka holmani (RRBP 10041, mid-trunk vertebra) from the late Oligocene Nsungwe Formation of Tanzania. (PDF) [file pone.0090415.s002.pdf]
